# Porcine Endogenous Retroviruses and Xenotransplantation, 2021

**DOI:** 10.3390/v13112156

**Published:** 2021-10-26

**Authors:** Joachim Denner

**Affiliations:** Department of Veterinary Medicine, Institute of Virology, Free University Berlin, 14163 Berlin, Germany; Joachim.Denner@fu-berlin.de; Tel.: +49-30-8386-3059

**Keywords:** porcine endogenous retroviruses, gammaretroviruses, xenotransplantation, virus safety

## Abstract

Porcine endogenous retroviruses (PERVs) are integrated in the genome of all pigs, and some of them are able to infect human cells. Therefore, PERVs pose a risk for xenotransplantation, the transplantation of pig cells, tissues, or organ to humans in order to alleviate the shortage of human donor organs. Up to 2021, a huge body of knowledge about PERVs has been accumulated regarding their biology, including replication, recombination, origin, host range, and immunosuppressive properties. Until now, no PERV transmission has been observed in clinical trials transplanting pig islet cells into diabetic humans, in preclinical trials transplanting pig cells and organs into nonhuman primates with remarkable long survival times of the transplant, and in infection experiments with several animal species. Nevertheless, in order to prevent virus transmission to the recipient, numerous strategies have been developed, including selection of PERV-C-free animals, RNA interference, antiviral drugs, vaccination, and genome editing. Furthermore, at present there are no more experimental approaches to evaluate the full risk until we move to the clinic.

## 1. Introduction

Xenotransplantation using pig cells, tissues, and organs is under development in order to alleviate the shortage of human donor organs. In the case of kidney allotransplantation, 40% of wait-listed patients are likely to die within five years, since chronic dialysis is a suboptimal form of therapy for many, and their quality of life remains poor [1]. Diabetes patients often suffer, due to insufficient compliance, from later complications despite insulin treatment. Since the supply of human pancreata or islet cells is very low [2], only xenotransplantation of pig islet cells may solve this problem. 

Pigs are suitable as donor animals, and they are advantageous due to their nearly unlimited availability, short breeding time, and large litters. Pig kidneys and hearts are comparable in size and function to human organs. There is a high physiological similarity, as, for example, pig insulin has been used to treat diabetes for decades. Pigs can be easily genetically modified and cloned, and pig farming is less expensive. In contrast, apes are endangered species, and most monkeys are too small to serve as donor animals.

Xenotransplantation must overcome three hurdles on its way into the clinic: rejection, especially hyperacute rejection (HAR); physiological incompatibility; and the risk of transmission of zoonotic pig microorganisms. HAR is based on the presence of preexisting antibodies against sugar residues present on pig proteins, but absent on human proteins. Zoonotic means that the pig microorganisms not only infect the transplant recipient, but induce a more or less severe disease. Whereas most pig viruses, bacteria, fungi, and protozoa can be eliminated by selection, vaccination, antiviral drugs, early weaning, colostrum deprivation, and embryo transfer, that is not possible for the porcine endogenous retroviruses (PERVs), because they are integrated in the genome of the pigs.

Several comprehensive reviews of xenotransplantation and PERVs have been published in the last years [3,4,5,6,7], and therefore the aim of this review is to give an overview of the topic for those who have never heard about xenotransplantation and PERVs, indicating the original publications and mainly previous reviews, as well as to give a detailed update on the data accumulated since the last reviews. 

## 2. Xenotransplantation: The Need and the Achievements

A high number of patients die on the waiting list, after not receiving a human donor organ. Concerning liver transplantation, in 2019, growth continued in the number of new waiting list registrations (12,767) in the United States; however, only 8896 transplants were performed, including living-donor transplants [8]. The situation is worse with other organs. In the United States, 3552 heart transplants were performed in 2019, but almost the same number of patients were waiting for a heart [9]. In the Eurotransplant region, 668 hearts were transplanted in 2019, but the active waiting list at the end of 2019 was 1119 [10], indicating that the demand for heart transplants far exceeds the number of donated human organs. In addition, Eurotransplant reported a decrease of transplanted organs from 50.8 to 47.4 transplants per million population of the member states per year [10], indicating a reduction of the number of available donors.

On this background, xenotransplantation offers several advantages over allotransplantation, among them nearly unlimited availability and increased microbiological safety. Since the pigs are generated under specified or designated pathogen-free conditions, the donor animals will be free of exogenous infectious microorganisms. In contrast, several viruses have been transmitted with solid organ allotransplantations, among them the human immunodeficiency virus (HIV), the rabies virus, the human cytomegalovirus (HCMV), Epstein-Barr virus, and others [11].

As a matter of fact, there are excellent achievements in the field of xenotransplantation, especially remarkable survival times of pig organ transplants in nonhuman primate recipients (Table 1). It is important to note that no PERV transmission was observed in these trials. In addition, the first clinical trials transplanting encapsulated pig islet cells into diabetic patients in New Zealand and Argentina and the preclinical trials with pig islet cells in nonhuman primates were successful [2,12,13]. Zhang and colleagues have recently completed a clinical trial of 47 patients suffering from fungal keratitis, all of whom were treated with decellularized corneal xenografts, with a success rate of 72% [14].

The excellent survival times were achieved due to numerous genetic modifications of the pigs, improved immunosuppression regimens, and removal of pathogenic viruses from the donor pigs. The first patient who received a human heart survived only 18 days [15]; the first patient in Germany, 27 h [16]. 

**Table 1:** Survival time of pig cells or organs in nonhuman primates.

Pig Transplant	Donor Pigs’ Genetic Background	Longest Survival Time (Days)	Reference
Islet cells	Wild-type minipigs	950	Shin et al. [17]
Heart, heterotopic	GTKO/CD46/TBM	945	Mohiuddin et al. [18]
Heart, orthotopic, life-supporting	GTKO/CD46/TBM	195	Längin et al. [19]
Kidney, life-supporting	GTKO/CD55	499	Kim et al. [20]
Neurons	CTLA4-Ig	549	Aron Badin et al. [21]
Cornea	GTKO	375	Yoon et al. [22]
Liver	GTKO	29	Shah et al. [23]
Lung	GTKO/CD47/CD55	14	Watanabe et al. [24]

CD46, cluster of differentiation 46, complement regulatory protein; CD47, integrin associated protein; CD55, complement decay-accelerating factor; CTLA4-Ig, cytotoxic T-lymphocyte-associated protein 4; GTKO, galactosyltransferase gene knockout; TBM, thrombomodulin.

## 3. Exogenous and Endogenous Retroviruses

Retroviruses are enveloped RNA viruses. They encode an enzyme called reverse transcriptase, which is able to transcribe their single-stranded RNA genome into a double-stranded DNA copy. Using another enzyme, the integrase, this DNA copy is integrated into the genome of the infected cell. The human immunodeficiency virus 1 (HIV-1), for example, infects CD4^+^ cells and integrates the viral DNA copy, which then is called a provirus, in the genome of these cells. No HIV-1 proviruses can be found, for example, in liver cells. HIV-1 is an exogenous retrovirus. When, however, a retrovirus infects and integrates into a sperm cell or an oocyte or into their precursor cells, after the fertilization of the oocyte by the sperm, the integrated retroviral provirus will be present in each cell of the developing embryo, and later of the whole organism. These integrated retroviruses in all cells of an organism are called endogenous retroviruses. Endogenous retroviruses are found in all reptiles, birds, and mammals, including humans. Most of the human endogenous retroviruses (HERVs) are defective due to mutations and deletions, only some; e.g., HERV-K, are able to produce viral particles that can be found in the placenta or in cells lines. In contrast to PERV, the HERV-K particles are not infectious. Antibodies against HERV-K have been found in tumor patients and pregnant women, indicating that virus proteins are expressed [25,26]. It is well known now that the envelope proteins of endogenous retroviruses of numerous species are functioning as syncytins in the placenta development (for review, see [27,28]). 

## 4. PERVs: Biology

PERVs are gammaretroviruses, previously classified by morphology as type C retroviruses, that are closely related to the murine leukemia virus (MuLV), feline leukemia virus (FeLV), and koala retrovirus (KoRV). MuLV, FeLV, and KoRV induce leukemia and immunodeficiency in their infected hosts [25,29]. Three subtypes of PERV have been named based on cell tropism, sequence variation, or receptor interference, as either PERV-A, PERV-B [30], or PERV-C [31], respectively. PERV-A and PERV-B are present in the genome of all pigs, while PERV-C is present in many, but not all pigs. PERV-A and PERV-B infect human cells and therefore pose a risk for xenotransplantation, while PERV-C infects only pig cells. However, in some pigs, PER-A/C recombinants were found, which were able to infect human cells and which were characterized by a high replication rates (see below). 

**Figure 1:**
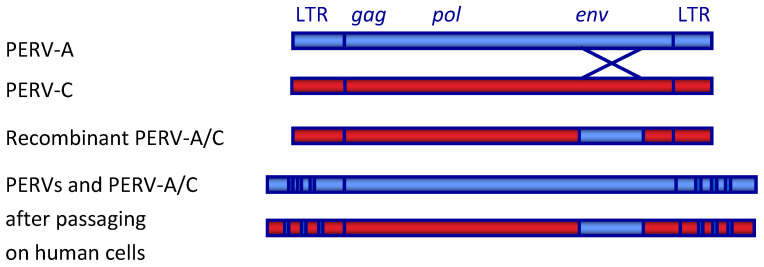
Schematic presentation of the genome of PERV. LTR, long terminal repeat; gag, group specific antigen; pol, polymerase; env, envelope. The recombinant PERV-A/C is the result of a recombination in the env gene spanning the receptor binding domain (RBD). During passaging of PERV-A and PERV-A/C on human cells, a multimerization of repeats in the LTR takes place [35].

The genes and open reading frames are typical for gammaretrovirus and have been described in detail [3] (Figure 1). The RNA genome encodes the core proteins (Gag, group-specific antigen), a polymerase (Pol) and other enzymes, and the envelope proteins (Env). 

The env gene codes for the surface (SU) envelope protein and the transmembrane (TM) envelope protein. The envelope proteins are responsible for binding to the cellular receptor and inducing membrane fusion. In the SU envelope protein, a receptor-binding domain (RBD) is located, binding to the receptor molecule. In the TM protein, a domain highly conserved among all retroviruses including HIV-1, the immunosuppressive (ISU) domain, was identified. Purified viruses, recombinant TM proteins, and synthetic peptides corresponding the ISU domain have been shown to inhibit lymphocyte stimulation and to modulate the cytokine release of lymphocytes (for review, see [32,33]). An immunosuppressive activity has also been shown for PERV [34]. The unique 3 (U3) and the unique 5 (U5) region, together with the repeat region (R) in the integrated provirus, constitute the so-called long terminal repeat (LTR): U3-R-U5. The LTRs contain binding sites for transcription factors, and viruses with LTRs containing more enhancer repeats are characterized by higher expression and replication [35]. 

## 5. Host Range In Vitro and In Vivo and Receptors

PERV-A and PERV-A/C are actually polytropic retroviruses, not only infecting human cells, but also cells of other species. A productive infection characterized by replication of PERV was observed for some immortalized human cell lines such as the kidney cell line 293, and cat cells (for review, see [3,36]). An infection without replication was observed for cells of minks, rhesus monkeys, baboons, gorillas, and chimpanzees. No infection was observed in the case of mouse, rat, rabbit, cotton rat, horse, pig-tailed macaque, African green monkey, and cynomolgus monkey cells. In contrast to human 293 cells, which allow production of PERV because they lost intracellular restriction factors [37], other human cell lines such as THP-1 and C8166 cells could be infected, but did not support PERV replication. Of interest are the infection of primary human cells. Endothelial cells, vascular fibroblast, and mesangial cells could be infected with PERV [38]. PBMC could only be infected with a human-cell-adapted PERV, characterized by a higher number of enhancer repeats inside the LTR of the virus [39]. However, it remains unclear in both cases whether the virus was produced. 

Based on these results, infection experiments in vivo were performed. Neither small animals nor nonhuman primates could be infected, even when pharmaceutical immunosuppression was applied (for review, see [3]). Only in the case of guinea pigs was a limited infection without evidence of replication observed in inoculated animals [40].

There are no new achievements in the field of viral receptors. The receptors for PERV-A (and PERV-A/C), are human porcine endogenous retrovirus-A receptor 1 and 2 (huPAR1 and huPAR2, respectively) [41]. They are members of the riboflavin transporter, also known as human riboflavin transporter 3 (hRFT3), and human riboflavin transporter 1 (hRFT1), respectively. More recently, these receptors have been renamed and classified as members of the solute carrier family of receptors, the “solute carrier family 52A” (SLC52A) [42]. The receptors for PERV-B and PERV-C are still unknown. 

The PERV receptor on baboon and other nonhuman primate cells was functional, but deficient by a mutation, explaining the low replication in these cells [43]. In mice, the receptor is mutated [44], explaining that mouse cells could not be infected, and infection experiments with high doses in vivo also failed [45]. Transgenic mice had been generated carrying the HuPAR-2, and it was reported that they could be infected with PERV [46], but no further investigation followed. Rats had only a low expression of the functional receptor, explaining that rat cells could not be infected; however, transfection with human or rat PAR-1 conferred susceptibility [44]. In summary, no animal models of PERV infection were found that would allow testing of antiretroviral drugs and vaccines.

## 6. The Origin of PERV

**Figure 2:**
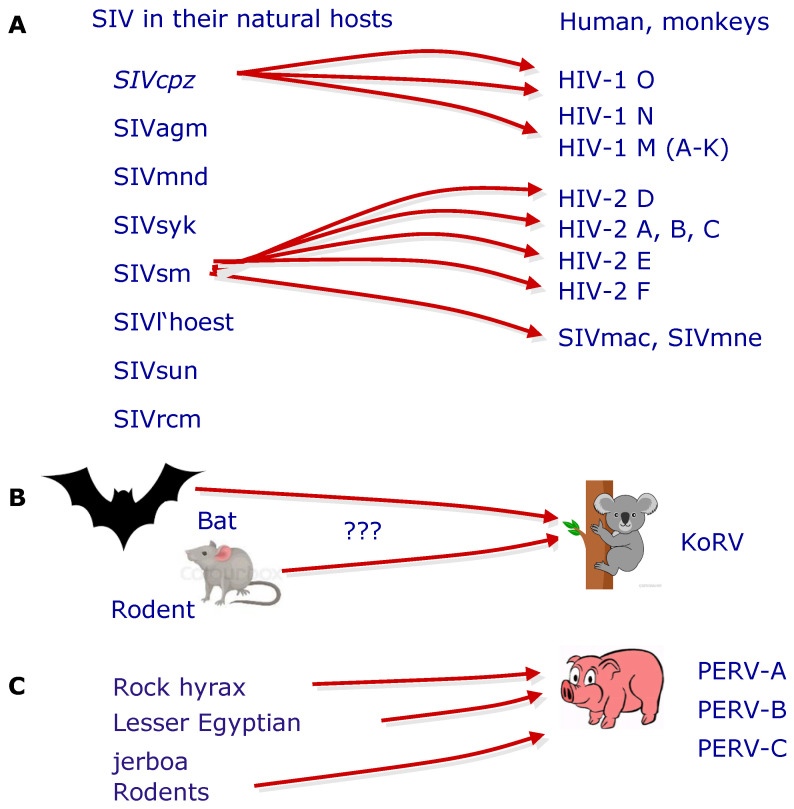
Examples of trans-species transmission of retroviruses. (**A**) Transmission of simian immunodeficiency viruses (SIV) from chimpanzee (cpz) or sooty mangabey (sm), which are apathogenic in their natural hosts, resulting in highly pathogenic human immunodeficiency viruses (HIV-1 and HIV-2). Different clades of HIV-1 and HIV-2 were described; all induce acquired immunodeficiency syndrome (AIDS). SIVsm also induces AIDS in rhesus monkeys (SIVmac) or Macaca nemestrina (SIVmne) [49,50,51]. (**B**) Example of a trans-species transmission of a gammaretrovirus, the koala retrovirus (KoRV), which is closely related to PERV, which induces lymphoma and immunodeficiency in koalas, and which was possibly derived from bats or rodents [52,53]. (**C**) Trans-species transmission from different species resulted in integrated PERVs in the pig genome [47,48].

PERV is the result of a trans-species transmission of a retrovirus or retroviruses from other species to the pig [47,48]. Trans-species transmission of retroviruses was and is a common mechanism of retrovirus distribution. The best investigated example is HIV [49,50,51] (Figure 2). Some gammaretroviruses, among them the KoRV, closely related to PERV, are also the result of a trans-species transmission [52,53] (Figure 2). PERVs are the result of a trans-species transmission of precursor retroviruses from different animals and further evolution in the pig genome. Ancestral PERV-like sequences were found in lesser Egyptian jerboas (*Jaculus jaculus*), in rock hyraxes (*Procavia capensis*), and in eight murid species, indicating an ancient trans-species transmission from non-porcine species to pigs [47,48] (Figure 2). 

## 7. Detection Systems

Numerous methods have been developed to detect PERV, both in the porcine donor and in the transplant recipient. These methods are methods that either directly detect viral RNA, proviral DNA, viral proteins, viral reverse transcriptase enzymatic activity, or infectious virus particles, or indirectly detect PERV-specific antibodies as sign of a viral infection. The detection methods, or better, the detection systems, which are defined as the complex of sample generation, sample preparation, sample origin, time of sampling, and the necessary negative and positive controls, along with the specific detection methods (either PCR-based, cell-based, or immunological methods), are well described in several reviews [3,54,55,56]. Of great importance for the evaluation of the safety of xenotransplantation is an assay detecting infectious viruses. At present, the most favored assay is based on infection of highly susceptible human 293 cells [57]; however, this assay is very insensitive, and more sensitive tests should be developed [58]. 

Further improvement of the detection systems and their application in virus elimination programs will lead to clean donor animals and a safe xenotransplantation. The detection of PERV is usually one part of strategies to screen for a broad spectrum of porcine microorganisms that may be zoonotic. Such comprehensive strategies and the tested microorganisms were described in detail [59,60,61,62,63,64]. New methods were added to the plethora of already described ones [65]. One of the new methods is droplet digital PCR (ddPCR), a method allowing to the correct measurement of the number of integrated proviruses.

## 8. Copy Number

The copy number of PERVs in the genome of pigs; e.g., the number of integrated proviruses, differs depending on the pig strain, the age of the animals, the organ analyzed, and the method used for detection (for review, see [66]). The PERV copy number per cell in Göttingen and Aachen minipigs as measured by ddPCR varies around 50 and 70 [59,67]; the number PERV copies of German landrace pigs genetically modified to be used in xenotransplantation and wild boars are in the same range, from 50 to 70 [67,68]. These are the copy numbers of integrated proviruses when analyzing high-molecular-weight DNA, not episomal DNA. Since retroviral DNA molecules are not able to replicate autonomously like episomes, they depend on integration for stable maintenance in cells [17]. The analysis of the copy number revealed that PERV is still active, and that the copy number increases during fetal development and after birth. Possibly, the generation of PERV-A/C recombinants, which were found in different organs, but never in the germ line of the pigs, may significantly contribute to the increase of the PERV copy number. The copy number of PERV proviruses was much lower in expanded potential stem cells (EPSCs) than in young and older pigs, confirming the increase in copy number during their lifetime [69].

## 9. PERVs and Restriction Factors 

The replication of a virus in host cells significantly depends on the presence or absence of cellular restriction factors. The biology of the restriction factors inhibiting PERV is well described [3,36,70]. APOBEC3 (apolipoprotein B mRNA editing enzyme, catalytic polypeptide-like 3G) is an effective inhibitor of PERV, and this explains why human 293 cells are highly susceptible to PERV, because these cells lost APOBEC3 [37]. In addition to APOBEC3, tetherins are good inhibitors of PERV release [70,71,72,73], and meanwhile it was shown that human tetherin was induced by interferon alpha (IFN-α). Consequently, an IFN-α treatment of 293T cells producing PERV reduced PERV release. The authors concluded that transgenic overexpression of tetherin may reduce the risk of PERV transmission in xenotransplantation. A combination of tetherin and APOBEC3 was shown to be more potent than each individual restriction factor [71]. SAM (sterile alpha motif) domain and HD (histidine (H) and/or aspartate (D)) domain-containing protein 1 (SAMHD1) is also an effective inhibitor of PERV by depleting the pool of dNTPs available to the reverse transcriptase for viral complementary DNA (cDNA) synthesis [74].

## 10. Recombinant PERVs and Minipigs 

In addition to PERV-A, PERV-B, and PERV-C, recombinants between PERV-A and PERV-C (PERV-A/C) were found in living animals. These recombinants were integrated only in the genome of somatic cells, but not in the germ line [57]. PERV-A/C acquired the receptor binding site for the PERV-A receptor, and therefore they are able to infect human cells and cells from other species. The recombinants have a higher replication rate compared with the paternal PERV-A [75], and there are several genetic elements responsible for their high infectivity [76]. It is important to note that the recombination sites in the envelope protein of PERV-A/Cs are different, indicating that the recombination is an individual event in each animal [59,76,77]. Minipigs are a unique pig breed concerning PERV-A/C, as they possess more copies of PERV-C sequences than many other pig breeds, and PERV-C is active in these pigs [76]. On the other hand, miniature swine that do not produce replication-competent PERV-C have been identified [78]. 

Based on these properties, PERV-A/C may pose a special risk to xenotransplantation [79], and therefore it is recommended to use PERV-C-free pigs for xenotransplantation, as they are unable to generate PERV-A/C [80].

**Figure 3:**
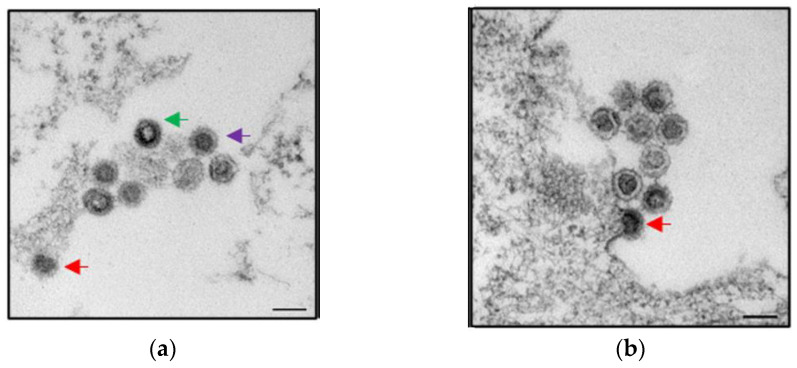
Two electron microscopic pictures (**a**,**b**) of recombinant PERV-A/C particles produced by human 293 cells. The virus was isolated from PBMCs of a Göttingen minipig and was able to infect 293 cells [59]. Budding viruses (red arrow), maturating viruses (green arrow), and mature viruses (lilac arrow) can be seen. Bar—200 nm. Courtesy of L. Möller and M. Laue, Robert Koch Institute, Berlin.

At present, PERV-A/C recombinants were only described—with one exception—for minipigs (for review, see [81]). The exception was US farm animals suffering from diseases, indicating that in diseased pigs, there is an increased incidence of PERV-A/C viremia [82]. PERV-A/C was detected in some of these diseased pigs over a long time. De novo infections and recombinations take place mainly in proliferating immune cells, because gammaretroviruses integrate only in proliferating cells. In diseased animals, which are setting up an effective immune response, the immune cells should proliferate massively. This assumption agrees with our finding that mitogen-stimulation of pig lymphocytes (of some kind simulating the immune stimulation) led to an increased expression of PERV [83,84,85]. Infectious replication competent PERV-A were also isolated from minipigs; for example, from Wuzhishan minipigs in China [86]. It is important to note that the probability of virus release is very low; in 11 Göttingen minipigs only in one case an infectious PERV-A/C could be isolated (Figure 3) [59]. Furthermore, animals not transmitting PERVs to human cells were identified in Massachusetts General Hospital (MGH) miniature swine [78].

## 11. PERVs and Stem Cells

Endogenous retroviruses have been found highly expressed in embryonic stem cells (ESCs) and induced pluripotent stem cells (iPSCs) of humans and mice, and they were used as markers for pluripotency [87,88,89,90]. A high expression of PERV was also observed in pig iPSCs [91]. Therefore, it was very surprising to see that in expanded potential stem cells (EPSCs), the expression of PERV was extremely low [69]. These cells were shown to express key pluripotency genes, to be genetically stable, and to differentiate to derivatives of the three germ layers, and additionally to trophoblast [92]. Therefore, EPSCs represent a unique state of cellular potency. 

## 12. PERVs and Pig Tumors 

Endogenous retroviruses were often found highly expressed in murine and human tumors; for example, the human endogenous retrovirus-K (HERV-K) was found expressed in human melanomas [93], prostate cancer [94], and other human tumors (for review, see [95,96]). It remains unclear whether the endogenous retrovirus contributes to the tumor development itself, or whether it is expressed due to transcriptional activation in the tumor cells. PERV particles were released from transformed pig kidney cells and lymphoma cells (for review, see [3]). PERV was found highly expressed in melanomas of melanoma-bearing MMS Troll pigs [97]. On the other hand, no PERV expression was found in two newly established pig lymphoma cell lines and L23 pig lymphoma cells [98]. Integrated, but not expressed, PERV-A/C recombinants were found only in the genome of L23 cells. Since in all three lymphoma cell lines the expression of PERV was very low, it seems unlikely that PERVs were involved in the pathogenesis of these lymphomas. However, all three lines were infected with the porcine lymphotropic herpesvirus-3 (PLHV-3), which may have been involved in lymphoma development. 

## 13. Absence of PERV Transmission in Preclinical and Clinical Trials 

In all preclinical and clinical trials performed until now, no PERV has been transmitted to the recipients. In the past, more than 200 humans have received a xenotransplantation product comprising pig cells, or tissues including ex vivo perfusion of pig organs or pig cell-based bioreactors (for review, see [3] and [99]). In the best documented human trials, encapsulated islet cells from Auckland Island pigs were transplanted to diabetic patients, and no PERV transmission was observed using both PCR-based and immunological methods [100,101,102]. 

Concerning the preclinical trials, in recent studies transplanting islet cell in marmosets [103] and cynomolgus monkeys [104], no PERV transmission was observed (Table 2). No PERV transmission was observed in a preclinical trial transplanting pig hearts from genetically modified pigs to baboons, with survival times of 182 and 195 days [26,105] (Table 2). These long survival times were achieved because in addition to an improved immunosuppressive regimen, non-ischaemic preservation with continuous perfusion, and control of post-transplantation growth of the transplant, the transmission of the porcine cytomegalovirus (PCMV) was prevented [105]. In this study, it was shown that no PERV was transmitted to the transplant recipient, although the donor pigs were positive for PERV-A, PERV-B, and PERV-C. PERV-A/C were not found in the donor pigs. In cases where PCMV was transmitted to the baboon recipient, and the survival time of the transplant was significantly reduced, PERV transmission also was not observed [105].

When analyzing streptozotocin-induced diabetic cynomolgus macaques that received porcine islet macrobeads implanted intraperitoneally, no PERV transmission was observed when their PBMCs were screened by PCR [63] (Table 2). The donor pigs were Large White-Yorkshire × Landrace F1 hybrid animals, and they were PERV-A, PERV-B, and PERV-C positive.

Regarding the cornea, transmission of PERV was not evident in both in vitro [107] and in vivo corneal transplantation studies [106,107]. In patients, PERV was not detectable up to 3.2 years after transplantation. 

Many other preclinical trials transplanting pig hearts, kidneys, islet cells, and cornea have been performed using effective immunosuppression regimens [27,63,108,109,110,111,112]. Unfortunately, in these studies, PERV transmission was not analyzed, but at least no clinical signs of a retrovirus infection were observed.

## 14. Strategies to Prevent PERV Infection 

In order to prevent transmission of PERV to the recipient, a range of different strategies have been developed. These strategies include the selection of PERV-C-free animals using specific and sensitive methods to detect PERV-C [113,114]. This prevents recombination between PERV-A and PERV-C. Other strategies are the selection of animals with a low expression of PERV-A and PERV-B [83], the generation of transgenic pigs expressing a PERV-specific small-interfering (si) RNA that reduces the expression of PERV [115,116,117,118,119], the development of a vaccine inducing neutralizing antibodies against the envelope proteins of PERV [120,121,122,123,124], and gene editing to inactivate all proviral copies in the genome using either a zinc finger nuclease [125] or the CRISPR/Cas9 (clustered regularly interspaced short palindromic repeats (CRISPR)/CRISPR-associated protein 9) technology [126,127].

### 14.1. Selection of Suitable Pigs

As shown, in PERV-C-positive animals, a recombination between PERV-A and PERV-C may happen, leading to a high-titer virus, and therefore it is recommended not to use PERV-C-positive animals. This is possible, since not all pigs carry PERV-C proviruses. In addition, it would be rational to use animals with a low expression of PERV-A and PERV-B, because the lower the expression at the RNA level, the lower the probability of producing protein and infectious virus particles. The expression of PERV differs significantly between animals of one breed and between different breeds [83,84,85]. Sensitive methods were developed to screen for PERV-C [113,114], and an assay based on mitogen stimulation of PBMCs helps to discriminate between pigs with high or low expression of PERV [83,84,85]. 

### 14.2. Antiretroviral Drugs

Antiretroviral drugs that also inhibit HIV-1, such as AZT (azidothymidine), have been found to inhibit PERV in vitro [128,129,130,131,132,133]. Until now, no one has investigated their activity in vivo, in the living pig; for example, in order to determine whether the antiretroviral drugs prevent generation of PERV-A/C recombinants in vivo. However, since it is recommended to use PERV-C-free animals in order to prevent recombination with PERV-A, this is only an academic question. The antiretroviral drugs can be used in case an infection of the recipient has taken place. However, the experience with the treatment of acquired immunodeficiency syndrome (AIDS) demonstrated that a monotherapy with a single antiviral may soon lead to resistance [134]. In this case, a combination therapy should be developed.

### 14.3. Vaccination

Whereas there is no vaccine against the retrovirus HIV-1, there are effective vaccines against different gammaretroviruses. Commercial vaccines against the FeLV, closely related to PERV, are on the market [135], and experimental vaccines against the murine leukemia virus, also closely related to PERV, have been developed [136,137]. Using the recombinant surface envelope and transmembrane envelope proteins of PERV, neutralizing antibodies were induced in several animal species, suggesting that such antibodies could also be induced in humans [120,121,122,123,124]. The combination of both proteins as ingredients in one vaccine resulted in higher titers of neutralizing antibodies compared with each envelope protein in a single application [122]. Because there is no animal model to test such vaccines against PERV, the corresponding transmembrane and surface envelope protein of the related FeLV were used to induce neutralizing antibodies against FeLV (for review, see [138]). Using this vaccination strategy, strong neutralizing antibodies binding to similar epitopes, as in the case of PERV, were induced, and cats could be protected from FeLV disease [139].

It is important to note that pigs do not produce antibodies against the surface and transmembrane envelope proteins of PERVs [140,141], indicating that the animals were tolerant and recognized these proteins as “self” in their ontogenesis. Why the animals produce antibodies against the core protein p27GAG at the same time remains unclear [141].

### 14.4. RNA Interference

At a time when no CRISPR/Cas systems were available, the best suited method to decrease PERV expression, and therefore to reduce the probability to release of infectious particles was RNA interference. Two laboratories used this method, and showed that the expression of PERV in vitro, in human cells producing PERV, and in vivo, in transgenic pigs expressing the PERV-specific shRNA, was reduced [115,116,117,118,119]. 

### 14.5. Genome Editing

Genome editing is a powerful tool to inactivate single genes in cells and animals [142]. The situation with PERV is more complicated, as it is integrated 50–70 times in the genome of a cell. Before the age of CRISPR/Cas systems, a zinc finger nuclease (ZFN) designed to bind specifically to sequences in the polymerase gene was used to inactivate all PERVs in human cells infected with PERV or pig PK15 cells producing PERV [125]. A highly conserved target sequence in the polymerase of all known proviruses was selected that should inactivate all PERVs in the genome. Expression and transport of the ZFN into the nucleus was shown by Western blot analysis, and by colocalization analysis, proximity ligation assay (PLA), and Förster resonance energy transfer (FRET) measurement. Unfortunately, the high expression of the ZFN was toxic to the transfected cells, most likely due to the specific cutting of the high copy number of the PERV proviruses [125].

**Figure 4:**
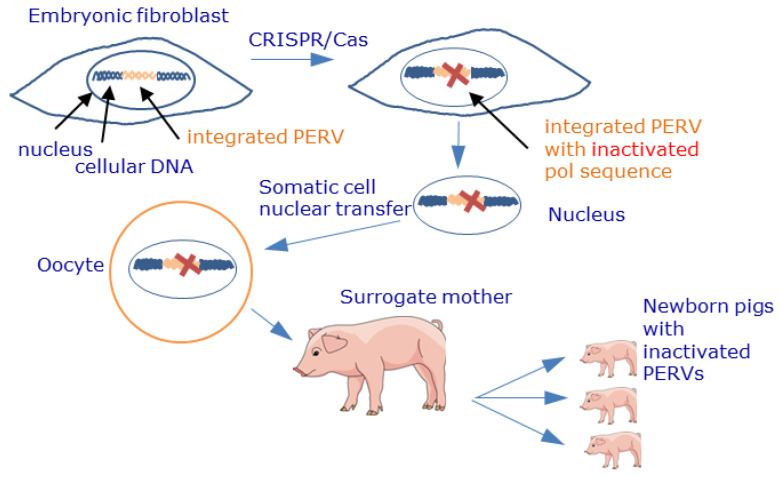
Schematic presentation of the inactivation of PERV proviruses by CRISPR/Cas and generation of piglets with inactivated PERV sequences. Using CRISPR/Cas, the PERV polymerase gene was inactivated, thus preventing the release of infectious viruses. The nuclei of these treated cells were transferred into pig oocytes, giving rise to embryos that were then transferred to surrogate sows. This process resulted in the birth of healthy piglets with inactivated PERVs [126,127].

The CRISPR/Cas technology also targeting the polymerase gene allowed the inactivation of all 62 PERV sequences in PK15 cells [126] as well as all 25 copies in embryonic cells used for the generation of newborn pigs [127] (Figure 4). Interestingly, the CRISPR/Cas9-treated PK15 cells still produced virus particles of the correct size; however, they were not infectious [143]. The altered morphology was possibly an off-target effect on the Gag protein or protease. The possibility of gene editing resulting in inactivated PERVs raised the question of whether conventional pigs can still be used for xenotransplantation, or whether only CRISPR/Cas9 inactivated pigs must be used as source animals for future xenotransplantations [144,145,146,147,148].

The following data support the view that CRISPR/CAS-treated animals may not be necessary:

1. As demonstrated above, until now in all clinical trials, among them transplantations of pig islet cells from Auckland Island pigs in diabetic patients in New Zealand and Argentina, no transmission of PERV was observed [3,99,100,101,102].
2. Furthermore, in all preclinical trials in nonhuman primates, no transmission of PERVs was observed [149,150,151,152]. However, nonhuman primates are not an ideal animal model to assess the risk of PERV transmission in xenotransplantation [153]. This is based on the fact that the main receptor for PERV-A and PERV-A/C entry, PERV-A receptor 1 (PAR-1), was found to be genetically deficient in baboons and cynomolgus monkeys (see above) [43]. In infection experiments in small animals and nonhuman primates with or without pharmaceutical immunosuppression, PERV transmission also was not observed: The mouse receptor was mutated and not effective, and the rat receptor was expressed only at low concentrations on the cell surface [44], showing that mouse and rat cells could not be infected [45,46,154].
3. Another problem is the potential off-target effects of CRISPR/Cas [155,156]. Off-target effects by CRISPR/Cas9 may occur, but they should be detected when analyzing the health of the animals and the functionality of the organs to be used for xenotransplantation.
4. The main obstacle is certainly the risk of inbreeding of CRISPR/Cas-inactivated pigs when generating high numbers of donor pigs for xenotransplantation.

## 15. Conclusions

Xenotransplantation using pig cells, tissues, or organs is regarded as the next great medical revolution [157]. PERVs are integrated in the genome of all pigs; they can be released as infectious particles, and some of them can infect human cells, therefore posing a risk for xenotransplantation. PERVs are typical gammaretroviruses, closely related to viruses inducing leukemia and immunodeficiencies in their hosts. They are, like many other endogenous and exogenous retroviruses, the result of a trans-species transmission. PERVs are still active in the living pig; the number of proviruses increases with age, and recombinant PERV-A/C integrate de novo into the genome of somatic cells. Accumulated knowledge of the biology, replication, release, and mutation of PERV, as well as numerous preclinical and clinical trials, allow for a better risk evaluation; however, there are no more experimental approaches to evaluate the full risk until we move to the clinic. To prevent PERV transmission, numerous strategies have been developed, including selection of PERV-C-free animals, RNA interference, antiviral drugs, vaccination, and genome editing, all of which can be applied in clinical trials. 

## Figures and Tables

**Table 2:** Recent preclinical xenotransplantations with reported PERV testing.

Recipient	Pig Transplant	Immunosuppression	PERV Screening		Reference
			In the Donor Pig	In the Recipient	
Rhesus monkeys	Fresh or decellularized cornea from SNU minipigs	Methylprednisolone, IVIG, Anti-CD40, or anti-CD154	PERV	No (PCR *)	[106]
Baboon	Orthotopic heart from GTKO, CD46, hTM pigs	anti-CD20, anti-CD40, MMF, methylpredni-solone	PERV-A, PERV-B, PERV-C	No (PCR)	[105]
Streptozoto-cin-induced diabetic cynomolgus macaques	Encapsulated islets from Large White-Yorkshire × Landrace F1 hybrids	None	PERV-A, PERV-B, PERV-C	No (PCR)	[63]
Nondiabetic cynomolgus macaques	Göttingen minipigs	None	PERV-A, PERV-B, PERV-C	No (PCR, Western blot)	[104]
Normo-glycaemic marmosets	Islet cells from German landrace hybrid pigs expressing LEA29Y	None	PERV-A, PERV-B, PERV-C	No (PCR, Western blot)	[103]

* The method used to detect PERV or anti-PERV antibodies is shown in parentheses. hTM, human thrombomodulin; IVIG, intravenous immunoglobulin; LEA29Y, T-cell costimulation inhibitor CTLA-4Ig; MMF, mycophenolate mofetil; SNU, Seoul National University.
